# Supplementary material for: Harnessing Greater Statistical Power: Comprehensive Evaluation of Disease Modifying Treatment Effects Across All or Multiple Post-Baseline Visits Compared to the Last Visit for Alzheimer’s Disease Clinical Trials
Source: medRxiv. 2025 Feb 21:2025.02.18.25322498. Preprint. [Version 1] doi: 10.1101/2025.02.18.25322498 (PMC11875270; doi:10.1101/2025.02.18.25322498)
Supplement: 1 [file NIHPP2025.02.18.25322498V1-supplement-1.pdf]

medRxiv preprint doi: https://doi.org/10.1101/2025.02.18.25322498; this version posted February 21, 2025. The copyright holder for this preprint (which was not certified by peer review) is the author/funder, who has granted medRxiv a license to display the preprint in perpetuity.
It is made available under a CC-BY 4.0 International license .

Supplemental Materials

Supplemental Table 1: Proportional treatment effect (i.e., % reduction) at each visit of the TRAILBLAZER-ALZ 2 Donanemab Phase 3 Trial (low/medium tau population) in CDR-SB

Weeks	Estimated % Reduction	SE	95% CI	
			Lower	Upper
12	36.4%	24.1%	-10.9%	83.7%
24	48.1%	11.8%	25.0%	71.3%
36	51.3%	8.4%	34.8%	67.9%
52	43.9%	7.0%	30.1%	57.8%
64	38.3%	6.7%	25.2%	51.5%
76	36.2%	5.9%	24.6%	47.8%
% difference between Weeks 36 and 12	14.9%	20.0%	-24.3%	54.2%
% difference between Weeks 36 and 64	13.0%	7.1%	-1.0%	27.0%
% difference between Weeks 36 and 76	15.2%	8.0%	-0.5%	30.8%

These results were generated using simulated semi-real trial data, replicating the observed disease progression trajectory in the low-to-medium tau population of the TRAILBLAZER-ALZ 2 donanemab trial[3]

Supplemental Table 2: Variance-covariance matrix and mean change from baseline (CFB) of CDR-SB extracted from Clarity-AD trial

Parameter	Months	3	6	9	12	15	18
Variance	3	0.72	0.83	0.82	0.76	0.68	0.60
Variance	6	0.83	1.50	1.48	1.37	1.23	1.08
Variance	9	0.82	1.48	2.28	2.12	1.90	1.66
Variance	12	0.76	1.37	2.12	3.06	2.75	2.41
Variance	15	0.68	1.23	1.90	2.75	3.85	3.38
Variance	18	0.60	1.08	1.66	2.41	3.38	4.63
Mean CFB	Placebo	0.36	0.61	0.80	1.16	1.41	1.66
	Lecanemab	0.27	0.43	0.58	0.81	1.02	1.21

Supplemental Table 3: Variance-covariance matrix and mean change from baseline (CFB) of CDR-SB extracted from the low-to-medium tau population of the TRAILBLAZER-ALZ 2 Trial

Parameter	Weeks	12	24	36	52	64	76
Variance	12	1.76	1.59	1.40	1.25	1.07	0.90
Variance	24	1.59	2.24	1.98	1.76	1.50	1.28
Variance	36	1.40	1.98	2.72	2.42	2.07	1.76

medRxiv preprint doi: https://doi.org/10.1101/2025.02.18.25322498; this version posted February 21, 2025. The copyright holder for this preprint (which was not certified by peer review) is the author/funder, who has granted medRxiv a license to display the preprint in perpetuity.
It is made available under a CC-BY 4.0 International license .

Variance	52	1.25	1.76	2.42	3.37	2.88	2.45
Variance	64	1.07	1.50	2.07	2.88	3.85	3.27
Variance	76	0.90	1.28	1.76	2.45	3.27	4.34
Mean CFB	Placebo	0.28	0.64	0.95	1.35	1.53	1.88
	Donanemab	0.18	0.33	0.46	0.75	0.94	1.20

Supplemental Table 4: Variance-covariance matrix and mean change from baseline (CFB) of iADRS extracted from the low-to-medium tau population of the TRAILBLAZER-ALZ 2 Trial

**Parameter**	**Weeks**	**12**	**24**	**36**	**52**	**64**	**76**
Variance	12	77.07	63.73	52.59	43.73	35.94	29.50
Variance	24	63.73	82.34	67.95	56.50	46.44	38.12
Variance	36	52.59	67.95	87.61	72.85	59.88	49.15
Variance	52	43.73	56.50	72.85	94.65	77.80	63.86
Variance	64	35.94	46.44	59.88	77.80	99.92	82.02
Variance	76	29.50	38.12	49.15	63.86	82.02	105.19
Mean CFB	Placebo	-0.91	-1.79	-3.29	-5.26	-7.03	-9.61
	Donanemab	0.48	0.30	-0.94	-2.74	-3.50	-5.81
